# Atypical Posterior Reversible Encephalopathy Syndrome in a Postpartum Woman With Moyamoya Disease: A Case Report and Literature Review

**DOI:** 10.3389/fneur.2021.696056

**Published:** 2021-09-16

**Authors:** Ning Zou, Guixiang Guo, Fangchao Wan, Xin Li

**Affiliations:** Department of Neurology, The First People's Hospital of Changde City, Changde, China

**Keywords:** postpartum woman, reversible posterior leukoencephalopathy syndrome, cerebrovascular occlusive disease, neurocognitive, differential diagnosis

## Abstract

**Background:** Moyamoya disease is a rare cerebrovascular occlusive disease, which is characterized by stenosis and gradual occlusion of the internal carotid arteries, causing the progression of characteristic collateral vessels. To date, most studies investigating moyamoya disease have focused on medical implications, and the potential implications for neurocognitive and/or neuropsychiatric functioning were inconclusive.

**Case Presentation:** we present a case of a 26-year-old Chinese postpartum woman who presented to the emergency department with a 19-h history of cognitive decline, vomiting, and convulsions. Blood pressure, heart rate, and respiration rate were 200/120 mmHg, 115 beats/minute, and 30 breaths/minute, respectively, on arrival. The Glasgow Coma Scale, modified RANKIN scale (mRS), and National Institute of Health stroke scale (NIHSS) scores were 3, 5, and 18, respectively. Moyamoya disease was diagnosed using cerebral angiography and digital subtraction angiography. The cognitive functions of orientation, use of language, ability to calculate, and memory significantly improved after 11 days of treatment (Glasgow Coma Scale: 15; mRS: 0; NIHSS: 0).

**Conclusions:**This patient was diagnosed with reversible posterior leukoencephalopathy syndrome related to moyamoya disease. This case highlights that atypical posterior reversible encephalopathy syndrome can occur in patients with moyamoya disease, and should be considered for the differential diagnosis of cerebral infarcts and hemorrhage in a postpartum female.

## Introduction

Moyamoya disease is characterized by stenosis or occlusion of the terminal portion of the internal carotid arteries and the branches in the circle of Willis. The etiology of moyamoya disease remains unclear; it can be observed idiopathically or secondary to other diseases. The diagnosis of moyamoya disease based on moyamoya vessels occurred bilaterally (1). Moreover, the angiographic image of moyamoya disease resembles a puff of smoke rifting in the air; the word “moyamoya” means “puff of smoke” in Japanese (2). A study reported that the prevalence of moyamoya disease was the highest in East Asia (0.35–0.94 per 100,000 of Japanese and Koreans), and the peak ages for moyamoya disease incidence were around 5 years-of-age and 40 years-of-age (2). In addition, Guey et al. reported that moyamoya disease is twice as prevalent in females as males (3). Nearly 10% of patients with moyamoya disease present as having a familial occurrence (4). Alterations in cognitive functioning and neurological symptoms are commonly observed in emergency departments (5). While some may have clear etiology, other less common etiologies must be considered. Therefore, additional laboratory testing and neuroimaging should be applied. This report reviews a rare case of a postpartum patient with moyamoya disease, who presented with altered cognitive function and neurological symptoms. This case highlights the importance of the awareness of uncommon presentations in postpartum patients leading to clinical suspicion of moyamoya disease.

## Case Report

**Figure 1:**
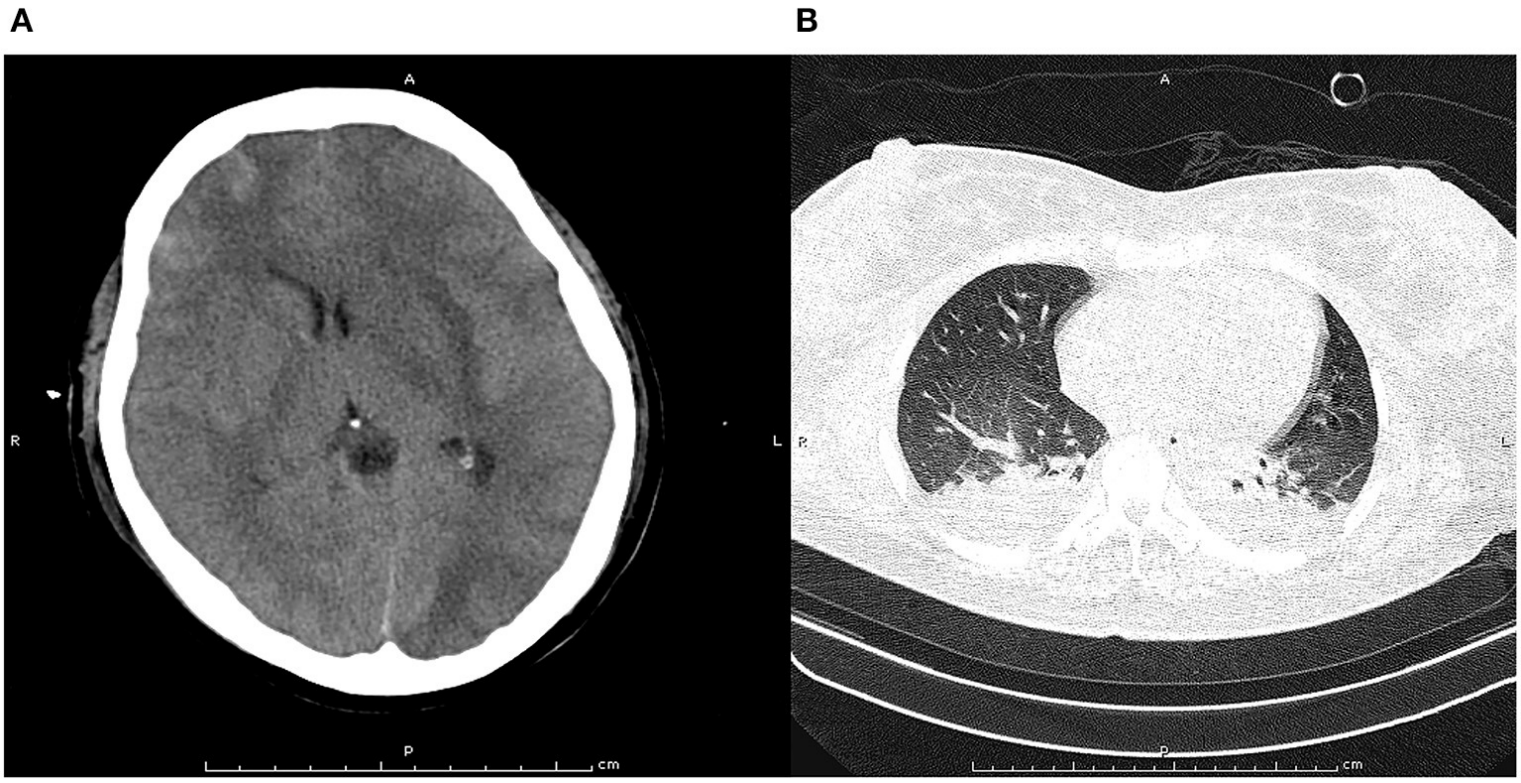
Pre-treatment computed tomography scan at cranial **(A)** and chest **(B)**. The pre-treatment computed tomography scan at cranial indicated obvious cerebral tissue edema, ventricle compression, and subarachnoid hemorrhage. The pre-treatment computed tomography scan at chest found bilateral infection with pleural effusion in lung.

**Figure 2:**
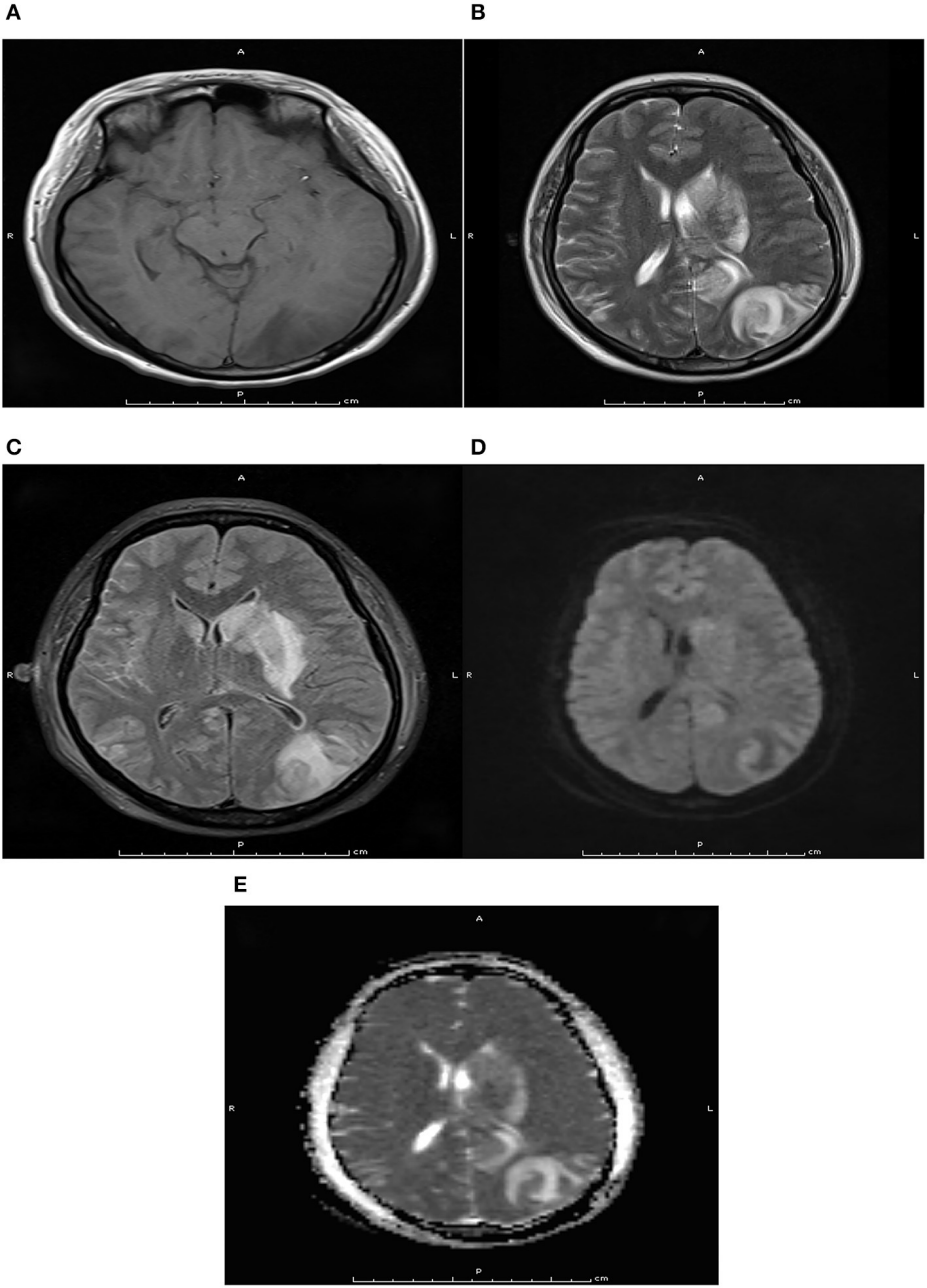
Pre-treatment axial magnetic resonance imaging at T1WI **(A)**, T2WI **(B)**, Flair **(C)**, DWI **(D)**, and ADC **(E)** sequence. Pre-treatment axial magnetic resonance imaging indicated there were long T1 and T2 signals, flair hypersignal, mild limited DWI, and ADC hypersignal in the left occipital lobe and basal ganglia.

**Figure 3:**
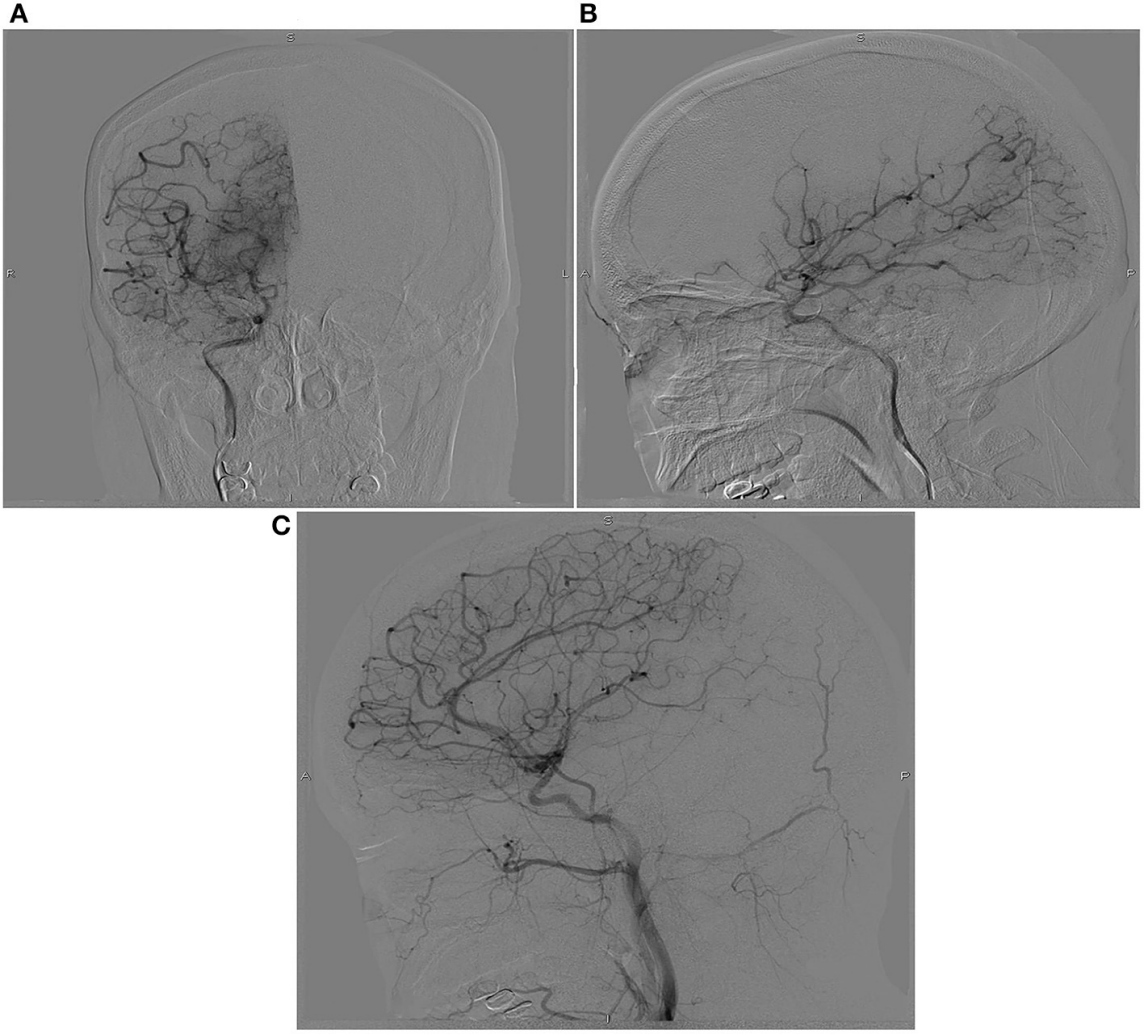
Pre-treatment digital subtraction angiography imaging at anterior **(A)**, posterior **(B)**, and lateral **(C)** projections. Pre-treatment digital subtraction angiography imaging indicated hyperplastic angiography at the end of the internal carotid artery, resembles a puff of smoke rifting in the air.

**Figure 4:**
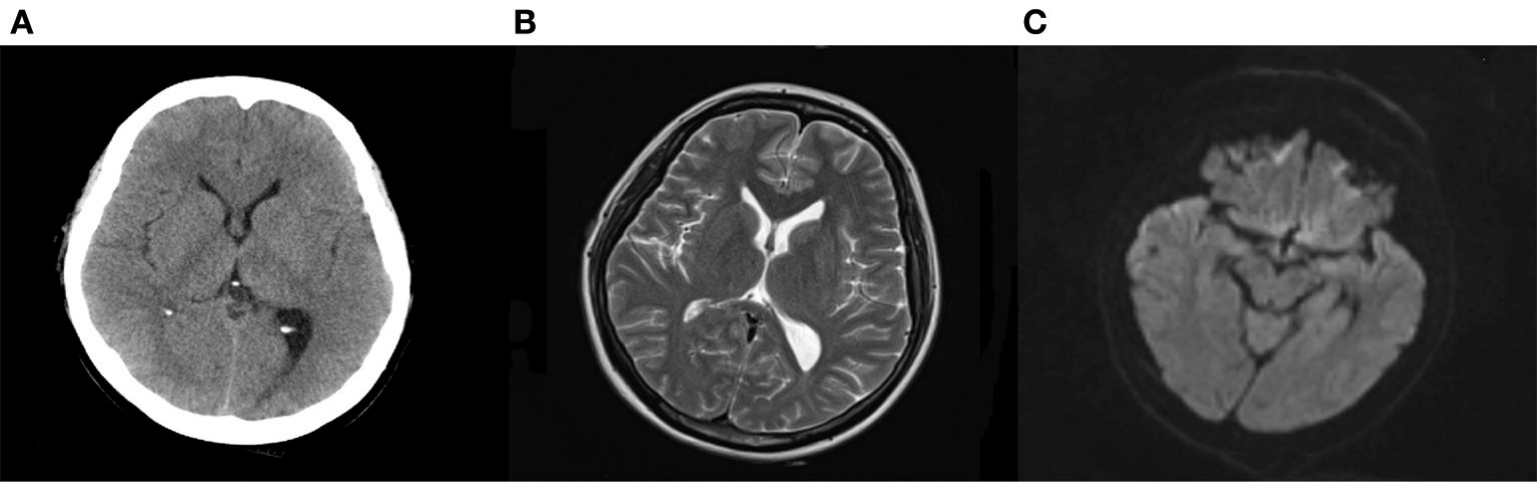
Computed tomography **(A)** and magnetic resonance imaging **(B,C)** after treatments. Computed tomography suggested the edema was disappeared, and the subarachnoid hemorrhage was absorbed after treatments. Magnetic resonance imaging found T2 hypersignal was disappeared, and the DWI signal without hypersignal shadow.

Written informed consent was obtained from the patient and ethical approval was granted by the Institutional Review Board of the First People's Hospital of Changde City (2017SK51308). A 26-year-old postpartum woman was admitted to the emergency department presenting with a 19-h history of cognitive decline, vomiting, and convulsions. She had no history of smoking, diabetes mellitus, hypertension, use of aspirin, or use of any recreational drugs. Blood pressure, heart rate, and respiration rate were 200/120 mmHg, 115 beats/minute, and 30 breaths/minute, respectively. The pupils were 3 mm with slow response to light stimulus. A flexion posture was observed in the left limb after tingling, while no movement was detected in the right limb. An initial computed tomography scan showed large infarcts and subarachnoid hemorrhage in the left frontal, temporal, occipital, and basal ganglia areas of the brain (Figure 1). The Glasgow Coma Scale (6), modified RANKIN scale (mRS) (7), and National Institute of Health stroke scale (NIHSS) (8) scores were 3, 5, and 18, respectively. Laboratory tests revealed total bilirubin, alanine aminotransferase, and platelet count were 66.0 μmol/L, 286 U/L, and 49 × 109/L, respectively. This indicated that hemolysis, elevated liver enzymes, and low platelets syndrome (HELLP) was occurring. The initial axial cerebral magnetic resonance imaging showed an area of vasogenic edema within the cortex, basal ganglia, and the subcortical white matter of the parieto-occipital lobes (Figure 2). Cerebral angiography and digital subtraction angiography were performed and moyamoya disease was diagnosed based on results (Figure 3). Treatment strategies: included urapidil (150 mg), cefamandole nafate (1 g/8 h), compound mannitol (125 ml/6 to 12 h), glucose and sodium chloride injection (500 ml), potassium chloride injection (15 ml qd), pantoprazole sodium (40 mg qd), Xingnaojing injection (10 ml qd), levamlodipine benzenesulfonate (5 mg qd), valsartan (80 mg qd), and carvedilol (12.5 mg bid). After 11 days, the Glasgow Coma Scale, mRS, and NIHSS scores were 15, 0, and 0, respectively. The patient's cognitive functions of orientation, use of language, ability to calculate, and memory were significantly improved. Moreover, cerebral magnetic resonance images were normal after treatment (Figure 4). Hence, the cognitive and neurological symptoms in a patient with moyamoya disease led to the additional diagnoses of posterior reversible encephalopathy syndrome (PRES), and associated puerperal HELLP syndrome and subarachnoid hemorrhage.

## Discussion

PRES was first described in 1996 and is characterized by acute neurological symptoms, including headache, seizures, visual disturbances, and other focal neurological deficits (9). The severity and acuity of neurological symptoms vary, and PRES generally occurs with rapid onset. PRES has been considered as a dysregulated perfusion disorder, which could cause reversible vasogenic edema. This report presented the case of a postpartum woman with moyamoya disease in which HELLP syndrome and PRES were also observed. Moyamoya is a relatively rare disorder in clinical practice. The diagnosis of moyamoya disease is based on cerebral angiography and digital subtraction angiography. The characteristic angiography results for moyamoya disease was the distal internal carotid arteries and proximal circle of Willis vessels affected by bilateral stenosis, and caused the involvement of prominent collateral vessels. Moreover, the severity of vascular abnormality could staged for predicting further ischemic or hemorrhage risk (10).

In our patient, the potential cause of PRES may have been subarachnoid hemorrhage or eclampsia. The mechanisms for the progression of PRES could be explained by the following: the hyperperfusion and increased cerebral perfusion pressure could cause the breakdown of the blood-brain barrier, which causes the extravasation of plasma and macromolecules into the interstitial space through tight junction proteins (11). Moreover, the release of nitric oxide, thromboxane A2, or endothelin-1 from vascular endothelium could mediate cerebral vasospasm and elevate blood pressure, which both play an important role in cerebral autoregulation (12). Furthermore, hypertension is a mutual response to hyperperfusion.

The etiologies of PRES should be mentioned. First, renal injury is an independent risk factor for PRES, and can be explained by the disruption of the renin-angiotensin-aldosterone system (13). Second, individuals infected with COVID-19 could develop cerebrovascular autoregulation disorder, acute renal failure, acute hypertension, hypoxia, inflammation, and endothelial injury; a high prevalence of PRES has been found in COVID-19 infected individuals (14). Third, preeclampsia and eclampsia are significantly associated with PRES. The endothelial dysfunction, elevated blood pressure, thrombocytopenia, and proteinuria are regarded as important features for the progression of PRES in preeclampsia and eclampsia (15, 16). Fourth, patients with PRES always have autoimmune disorders, including systemic lupus erythematosus, thrombotic thrombocytopenic purpura, Crohn's disease, and scleroderma (17). Finally, the use of immunosuppression and several other medications are significantly associated with increased risk of PRES (18–20).

The clinical presentation of postpartum women with moyamoya disease varies. Maruyama et al. reported a 41-year-old postpartum woman suffering with sudden onset of dysarthria and left extremity weakness 6 days after delivery, and found protein Z deficiency and a hypercoagulation state which are both significantly associated with ischemic stroke in patients with moyamoya disease (21). Kakogawa et al. reported an antepartum intracranial hemorrhage caused by unilateral moyamoya disease (22). Park et al. reported that severely reduced regional cerebrovascular reserve and frequent transient ischemic attacks at antepartum might present as neurologic deterioration during pregnancy, delivery, and puerperium (23). Furthermore, postpartum women with moyamoya disease could experience seizures and subarachnoid hemorrhage (24). These results suggested the most common presenting symptoms for patients with moyamoya disease was ischemic attacks, and hemorrhagic forms were also observed for adults (25). The current report presents a postpartum woman with moyamoya disease, with related HELLP syndrome and PRES. A potential explanation could be that the baseline blood pressure in postpartum women is higher, and this may have contributed to the observed cognitive and neurological symptoms.

## Conclusions

This report presents a rare case of atypical PRES due to moyamoya disease in a patient with HELLP syndrome during the postpartum period. Therefore, moyamoya disease should be regarded as an underlying disease of PRES in postpartum women. However, this study based on single case description, and the association might due to chance. Clinicians should be familiar with the potential complications of moyamoya disease, particularly in pregnant women. The appropriate treatments should be utilized to manage moyamoya disease, and patients need to be closely monitored and referred to appropriate specialists.

## Data Availability Statement

The original contributions presented in the study are included in the article/supplementary material, further inquiries can be directed to the corresponding author/s.

## Ethics Statement

The studies involving human participants were reviewed and approved by the Institutional Review Board of the First People's Hospital of Changde City. The patients/participants provided their written informed consent to participate in this study. Written informed consent was obtained from the individual(s) for the publication of any potentially identifiable images or data included in this article.

## Author Contributions

GG and XL conceived the idea, conceptualized the study, and drafted the manuscript. NZ collected the data. FW analyzed the data. XL reviewed the manuscript. All authors read and approved the final draft.

## Funding

This work was funded by Hunan Provincial Department of Science and Technology (2017SK51308).

## Conflict of Interest

The authors declare that the research was conducted in the absence of any commercial or financial relationships that could be construed as a potential conflict of interest.

## Publisher's Note

All claims expressed in this article are solely those of the authors and do not necessarily represent those of their affiliated organizations, or those of the publisher, the editors and the reviewers. Any product that may be evaluated in this article, or claim that may be made by its manufacturer, is not guaranteed or endorsed by the publisher.
